# A Phospholipid-Protein Complex from Krill with Antioxidative and Immunomodulating Properties Reduced Plasma Triacylglycerol and Hepatic Lipogenesis in Rats

**DOI:** 10.3390/md13074375

**Published:** 2015-07-16

**Authors:** Marie S. Ramsvik, Bodil Bjørndal, Inge Bruheim, Pavol Bohov, Rolf K. Berge

**Affiliations:** 1 Department of Clinical Science, University of Bergen, Bergen N-5020, Norway; E-Mails: Marie.Ramsvik@olympic.no (M.S.R.); Bodil.Bjorndal@uib.no (B.B.); Pavol.Bohov@uib.no (P.B.); 2 Olympic Seafood AS, Fosnavåg N-6099, Norway; E-Mail: Inge.Bruheim@olympic.no; 3 Department of Heart Disease, Haukeland University Hospital, Bergen N-5021, Norway

**Keywords:** Antarctic krill, lipogenesis, plasma lipids, inflammation, antioxidant capacity, omega-3 polyunsaturated fatty acids, cholesterol, lipid lowering

## Abstract

Dietary intake of marine omega-3 polyunsaturated fatty acids (*n*-3 PUFAs) can change the plasma profile from atherogenic to cardioprotective. In addition, there is growing evidence that proteins of marine origin may have health benefits. We investigated a phospholipid-protein complex (PPC) from krill that is hypothesized to influence lipid metabolism, inflammation, and redox status. Male Wistar rats were fed a control diet (2% soy oil, 8% lard, 20% casein), or diets where corresponding amounts of casein and lard were replaced with PPC at 3%, 6%, or 11% (wt %), for four weeks. Dietary supplementation with PPC resulted in significantly lower levels of plasma triacylglycerols in the 11% PPC-fed group, probably due to reduced hepatic lipogenesis. Plasma cholesterol levels were also reduced at the highest dose of PPC. In addition, the plasma and liver content of *n*-3 PUFAs increased while *n*-6 PUFAs decreased. This was associated with increased total antioxidant capacity in plasma and increased liver gene expression of mitochondrial superoxide dismutase (*Sod2*). Finally, a reduced plasma level of the inflammatory mediator interleukin-2 (IL-2) was detected in the PPC-fed animals. The present data show that PPC has lipid-lowering effects in rats, and may modulate risk factors related to cardiovascular disease progression.

## 1. Introduction 

Health benefits of a diet rich in marine products have been demonstrated in several studies over the last decades. More specific, the marine polyunsaturated omega-3 fatty acids (*n*-3 PUFA) eicosapentaenoic acid (EPA, C20:5 *n*-3) and docosahexaenoic acid (DHA, C22:6 *n*-3) exhibit health-promoting effects in both basic research models and in clinical trials. The health benefits associated with intake of marine *n*-3 PUFA is best documented in the prevention of cardiovascular disease [1,2,3,4,5,6,7,8]. The preventive role is explained by their ability to lower plasma triacylglycerol (TAG) [9,10,11], reduce platelet aggregation [12,13] and blood pressure [14], protect against cardiac arrhythmias [15], and potentially reduce inflammation [16,17,18]. 

Recent scientific works also focus on the effect of marine proteins as potentially important components for human health [19,20,21]. In rat studies, diets with salmon protein have demonstrated lower weight gain associated with reduced visceral fat deposition [22]. Fish protein has also been shown to reduce plasma cholesterol by increasing fecal cholesterol and bile acid secretion [23], and influence hepatic expression of genes involved in lipid homeostasis in rats [24]. In addition, peptides isolated from a number of fish species have demonstrated antioxidant effects [25,26,27,28], and a salmon hydrolysate has been found to reduce plasma cytokine levels and atherosclerosis in apolipoprotein E-deficient (ApoE^−/−^) mice [29].

Antarctic Krill (*Euphausia superba*) is a zooplankton crustacean rich in protein and lipids [30]. In common with oily fish species, krill is a good source of *n*-3 fatty acids. A major part of the EPA and DHA in krill is in the form of phospholipids (PLs), whereas in fish oil they are in the form of TAG, or chemically synthesized to fatty acid ethyl esters. Studies in animals have suggested a higher bioavailability of *n*-3 PUFAs incorporated in PL compared to TAG [31,32,33], which is also reported from controlled human intervention studies [34], but these findings are still inconclusive [35]. In addition to its *n*-3 PUFA content, Antarctic krill is a source of high-quality marine proteins containing all essential amino acids [36]. We have previously shown that a krill powder containing 40% protein and 60% fat had a plasma lipid lowering effect and affected expression of hepatic genes involved in lipid- and glucose metabolism in mice [37]. Additionally, in mildly obese men, a 24-week krill powder treatment was shown to reduce TAG [38]. This may have been due to the high level of omega-3 PUFAs in the krill powder, but the combination of nutrients such as proteins, peptides, astaxanthin, and *n*-3 PLs may also act independently and/or synergistically to promote the biological response. The PPC investigated in the present study consisted of approximatly 46% protein and 46% fat, and was produced with enzymatic hydrolysis of fresh krill, at low temperature, to ensure low levels of oxidation and degradation of fatty acids. This is the first study that has been performed on this product.

The objective of this study was to investigate the effect of a PPC from krill on body composition, plasma lipid levels, and hepatic lipogenesis in a dose-dependent manner in male Wistar rats. In addition, the effect of PPC on inflammation and antioxidant capacity was evaluated. Based on the suggested role of inflammatory processes in the pathogenesis of atherogenesis, and of our previous demonstration of lipid-lowering effects of krill oil [31] and protein [37], any immunomodulating effects and/or effects on redox status of the investigated PPC would be of particular interest for the potential use of PPC in atherosclerotic disorders. 

## 2. Results

### 2.1. Animals and Diets 

**Figure 1:**
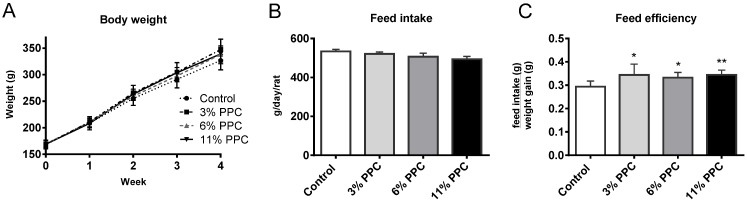
Weight gain and feed intake in male Wistar rats fed a protein-phospholipid complex (PPC) from krill. (**A**) Weekly weight development; (**B**) feed intake; and (**C**) feed efficiency in controls, and in the 3%-, 6%- or 11%-PPC supplemented group. Values are means with standard deviations (*n* = 6 for **A** and **C**, *n* = 3 for **B**). Significant difference from controls was determined using unpaired *t*-test (* *p* ≤ 0.05, ** *p* ≤ 0.01).

Male Wistar rats were randomly divided into four groups, and fed either a control diet (2% soy oil, 8% lard, 20% casein), or experimental diets where casein and lard were replaced with PPC at 3%, 6%, or 11% (wt %), for four weeks. All rats followed the same growth curve, with no differences in weight at baseline or at the end of the study (Figure 1A). Despite a similar weight gain in all groups, food intake tended to be lower in the PPC-fed groups resulting in a significantly higher feed efficiency (weight gain (g)/feed intake (g)) compared to controls (Figure 1B,C). 

The liver, heart, and four adipose tissue depots (mesenteric, epididymal, perirenal, and subcutaneous white adipose tissue depots) were dissected and weighed. There was no significant difference in dissection weights between the groups of any of these tissues (Figure S1). 

### 2.2. Plasma Lipids and Fatty Acid Composition 

**Figure 2:**
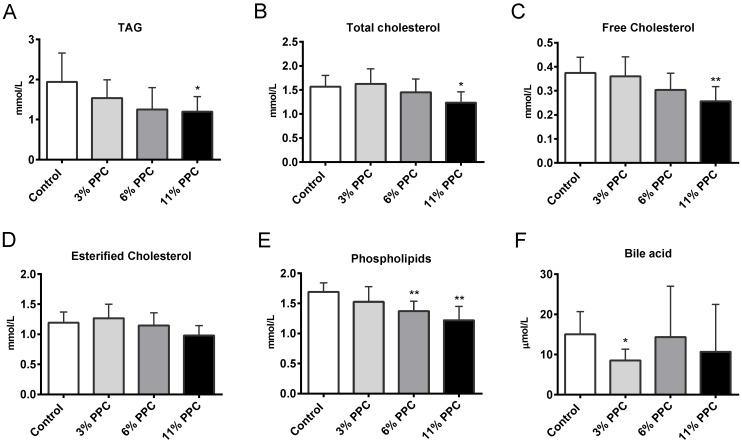
Plasma lipids in male Wistar rats fed a protein-phospholipid complex (PPC) from krill. (**A**) Triacylglycerol (TAG); (**B**) total cholesterol; (**C**) free cholesterol; (**D**) esterified cholesterol; (**E**) phospholipids and (**F**) bile acids were measured by specific enzymatic reactions. Values are means with standard deviations (*n* = 6). Significant difference from control was determined using unpaired *t*-test (* *p* ≤ 0.05, ** *p* ≤ 0.01).

Total plasma concentrations of TAG and cholesterol were significantly lower in the 11% PPC supplemented group compared to controls (Figure 2A,B). The decrease in total cholesterol was mainly due to lower levels of free cholesterol, while esterified cholesterol was less affected (Figure 2C,D). Plasma PL levels were significantly decreased by both the 6% and the 11% PPC supplemented diet (Figure 2E). The plasma levels of high-density lipoprotein (HDL) cholesterol, non-esterified fatty acids (NEFAs), glucose and insulin were not significantly affected by PPC, while low-density lipoprotein (LDL) cholesterol showed a small, but significant, increase in the 6% PPC group (Figure S2). Bile acid levels were significantly reduced only in the 3% PPC group (Figure 2F).

**Figure 3:**
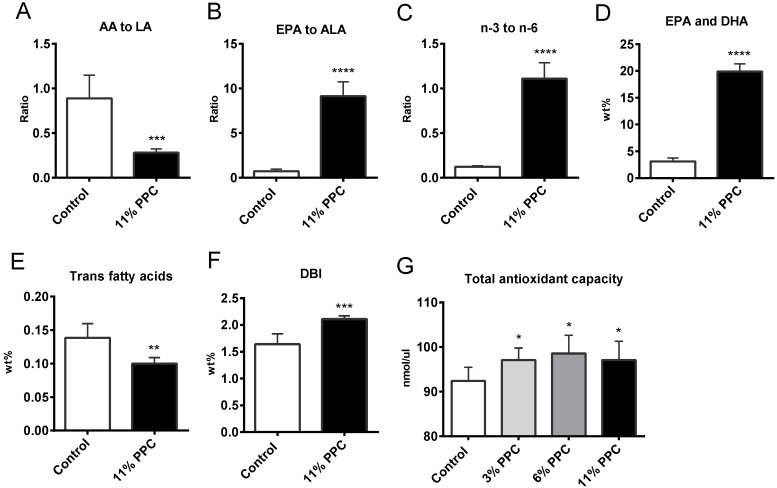
Plasma fatty acid concentration, ratios and indexes in male Wistar rats fed a protein-phospholipid complex (PPC) from krill. (**A**) Ratio between arachidonic acid (AA, C20:4*n*-6) to linoleic acid (LA, C18:2*n*-6); (**B**) ratio between eicosapentaenoic acid (EPA, C20:5*n*-3) to alpha linolenic acid (ALA, C18:3*n*-3); (**C**) ratio between *n*-3 to *n*-6 polyunsaturated fatty acids (PUFAs); (**D**) wt % of EPA + docosahexaenoic acid (DHA, C22:6*n*-3); (**E**) wt % of trans fatty acids; (**F**) Double bond index (DBI) (defined as the sum of all fatty acids with one or more double bonds/total fatty acids); and (**G**) total antioxidant capacity. Values are means with standard deviations (*n* = 6). Significant difference from control was determined using unpaired *t*-test (** *p* ≤ 0.01, *** *p* ≤ 0.001).

Plasma fatty acid composition was determined in controls and in the 11% PPC-fed group (Table 1). The wt % of total saturated fatty acids (SFAs) did not differ between the groups, neither were the individual shorter SFAs C10:0-C14:0, whilst a small, but significantly lower level of eicosanoic acid (C20:0) was observed in the PPC-fed group (Table 1). Although the wt % of total monounsaturated fatty acids (MUFAs) was not significantly lower in the PPC-fed group compared to controls, a significant lower level of oleic acid (OA, C18:1*n*-9) and eicosenoic acid (C20:1*n*-9) resulted (Table 1). Small differences were observed in the composition of the long-chain MUFAs, although the levels of erucic acid (C22:1*n*-9) increased significantly in the PPC-fed group (Table 1). The plasma levels of total PUFAs did not significantly differ after PPC feeding compared to control. However, the wt % of *n*-9 PUFAs were significantly reduced due to lower levels of mead acid (MA, C20:3*n*-9) in the PPC-fed group (Table 1). Most *n*-6 PUFAs were reduced by PPC, in particular arachidonic acid (AA, C20:4*n*-6), which was reduced by 71%. Linoleic acid (LA, C18:2*n*-6) was however unchanged, which led to a three-fold lower ratio of AA to LA (Figure 3A). All n-3 PUFAs increased significantly by the 11% PPC feeding, in particular EPA, which increased to a 24-fold higher level than in controls, increasing the ratio of EPA to alpha linolenic acid (ALA, C18:3*n*-3) 13-fold (Figure 3B). It was also of interest that the wt % of heneicosapentaenoic acid (HPA, C21:5*n*-3) increased 28-fold in the PPC-fed animals (Table 1). In total, this resulted in an increased *n*-3 to *n*-6 PUFA ratio, increased wt % of EPA and DHA, while plasma *trans* fatty acids were reduced (Figure 3C–E).

**Table 1:** Fatty acid compositions (wt %) in plasma of male Wistar rats ^1^.

	Diets		
	Control	11% PPC
**SFAs**	30.25 ± 0.69	29.07 ± 1.39
C10:0	0.01 ± 0.00	0.01 ± 0.00
C12:0	0.03 ± 0.00	0.03 ± 0.00
C14:0	0.65 ± 0.21	0.76 ± 0.12
C16:0	19.57 ± 1.18	19.91 ± 0.86
C18:0	8.58 ± 1.87	6.87 ± 0.95
C20:0	0.07 ± 0.01	0.05 ± 0.01 **
C22:0	0.10 ± 0.02	0.08 ± 0.01
**MUFAs**	26.69 ± 7.16	21.74 ± 2.91
C16:1*n*-7	3.09 ± 1.49	3.51 ± 1.27
C16:1*n*-9	0.36 ± 0.15	0.23 ± 0.04
C18:1*n*-7	3.51 ± 1.23	3.07 ± 0.54
C18:1*n*-9 (OA)	18.72 ± 4.32	13.94 ± 1.56 *
C20:1*n*-7	0.26 ± 0.09	0.24 ± 0.06
C20:1*n*-9	0.22 ±0.07	0.12 ± 0.03 **
C22:1*n*-7	0.04 ± 0.02	0.04 ± 0.01
C22:1*n*-9	0.01 ± 0.00	0.03 ±0.01 **
C24:1*n*-9	0.19 ± 0.06	0.21 ± 0.03
**PUFAs**	42.92 ± 8.20	49.08 ± 5.72
*n*-9 PUFAs	0.21 ± 0.03	0.09 ± 0.01 ***
C20:3*n*-9 (MA)	0.21 ± 0.03	0.09 ± 0.01 ***
*n*-6 PUFAs	38.10 ± 6.08	23.39 ± 2.51 ***
C18:2*n*-6 (LA)	18.97 ± 1.36	17.51 ± 1.84
C18:3*n*-6 (GLA)	0.28 ± 0.05	0.10 ± 0.01 ***
C20:3*n*-6 (DGLA)	0.79 ± 0.25	0.55 ± 0.04 *
C20:4*n*-6 (AA)	16.97 ± 5.51	4.90 ± 0.93 ***
C22:4*n*-6	0.44 ± 0.04	0.05 ± 0.01 ***
C22:5*n*-6 (DPA)	0.24 ± 0.05	0.06 ± 0.00 ***
*n*-3 PUFAs	4.62 ± 0.67	25.61 ± 1.75 ***
C18:3*n*-3 (ALA)	0.76 ± 0.09	1.41 ± 0.14 ***
C18:4*n*-3	0.03 ± 0.01	0.51 ± 0.07 ***
C20:4*n*-3	0.10 ± 0.01	0.54 ± 0.14 ***
C20:5*n*-3 (EPA)	0.54 ± 0.12	12.72 ± 1.41 ***
C21:5*n*-3 (HPA)	0.01 ± 0.00	0.28 ± 0.06 ***
C22:5*n*-3 (DPA)	0.62 ± 0.07	2.98 ± 0.41 ***
C22:6*n*-3 (DHA)	2.56 ± 0.53	7.17 ± 0.64 ***

^1^ Data were analyzed with *t*-test (*n* = 6), and are presented as means ± SD of wt % (g fatty acids/100 g total fatty acids). Values significantly different from control are indicated (* *p* ≤ 0.05, ** *p* ≤ 0.01, *** *p* ≤ 0.001). Abbrevations: AA, arachidonic acid; ALA, alpha linolenic acid; DGLA, dihomo-gamma-linolenic acid; DHA, docosahexaenoic acid; DPA, docopentaenoic acid; EPA, eicosapentaenoic acid; GLA, gamma-linolenic acid; HPA, heneicosapentaenoic acid; LA, linoleic acid; MA, meads acid; OA, oleic acid; PPC, phospholipid-protein complex.

### 2.3. Effect on Antioxidant Status

In agreement with an increased plasma double bond index (DBI) (Figure 3F), PPC seemed to have antioxidant potential as the plasma total antioxidant capacity (Figure 3G), and the hepatic gene expression of mitochondrial superoxide dismutase (*Sod2*) was significantly increased in the PPC-fed animals compared to controls (Table 2). 

**Table 2:** Hepatic gene expression in male Wistar rats ^1^.

		Diets			
Symbol	Function	Control	11% PPC		*p*-Value
*Srebf1*	Transcription factor	1.00 ± 0.35	0.77 ± 0.08		0.189
*Ppara*	Transcription factor	1.00 ± 0.34	1.65 ± 1.06		0.262
*Cd36/Fat*	Fatty acid import	1.00 ± 0.23	1.27 ± 0.51		0.339
*Cpt1a*	β-oxidation	1.00 ± 0.33	2.07 ± 1.22		0.092
*Cpt2*	β-oxidation	1.00 ± 0.33	2.05 ± 1.19		0.118
*Cact/Slc25a20*	β-oxidation (transport)	1.00 ± 0.19	1.53 ± 0.68		0.162
*Acox1*	β-oxidation	1.00 ± 0.09	1.65 ± 0.44		0.017
*Hmgcs2*	Ketogenesis	1.00 ± 0.23	1.38 ± 0.70		0.320
*Fasn*	Fatty acid synthesis	1.00 ± 0.71	0.37 ± 0.26		0.103
*Acaca*	Fatty acid synthesis	1.00 ± 0.53	0.71 ± 0.23		0.298
*Elo1*	Fatty acid elongation	1.00 ± 0.10	1.06 ± 0.27		0.690
*Scd1*	Δ9 desaturation	1.00 ± 0.61	0.94 ± 0.59		0.890
*Fads1*	∆5 desaturation of fatty acids	1.00 ± 0.18	0.53 ± 0.22		0.011
*Fads2*	Δ6 desaturation of fatty acids	1.00 ± 0.25	0.62 ± 0.24		0.061
*ApoB*	Cholesterol import	1.00 ± 0.10	1.06 ± 0.20	0.617	
*Ldlr*	Cholesterol import	1.00 ± 0.19	1.03 ± 0.37	0.898	
*Hmgcr*	Cholesterol synthesis	1.00 ± 0.23	1.07± 0.19	0.636	
*Cyp7a1*	Bile synthesis	1.00 ± 0.43	1.91 ± 1.64	0.304	
*Soat 1/Acat*	Cholesterol ester formation	1.00 ± 0.14	0.98 ± 0.31	0.888	
*Gpam*	TAG synthesis (glycerolipid synthesis)	1.00 ± 0.20	0.88 ± 0.18	0.386	
*Dgat*	TAG synthesis	1.00 ± 0.17	1.24 ± 0.26	0.166	
*Mttp*	Lipoprotein assembly	1.00 ± 0.11	1.20 ± 0.34	0.301	
*Lipc*	Hepatisk lipase	1.00 ± 0.13	0.98 ± 0.22	0.850	
*Aadac*	Triglycerid lipase activity	1.00 ± 0.11	1.20 ± 0.19	0.112	
*Sod2*	Mitochondrial antioxidant defense system	1.00 ± 0.14	1.37 ± 0.26	0.038	

PPC, phospholipid-protein complex. ^1^ All values were normalized to *Rplp0* and values relative to control are shown as means ± SD (*n* = 5 in control, *n* = 6 in 11% PPC). Results were analyzed by unpaired *t*-test, with significant *p*-values in bold (*p* ≤ 0.05). Abbreviations: *Aadac*, arylacetamide deacetylase; *Acaca*, acetyl-coA-carboxylase alpha; *Acox1*, acyl-CoA oxidase 1 palmitoyl; *ApoB*, apoprotein B; *Cact/Slc25a20*, carnitine-acylcarnitine translocase; *Cd36/Fat*, CD36 antigen/fatty acid translocase; *Cpt1a*, carnitine palmitoyltransferase 1A; *Cpt2*, carnitine palmitoyltransferase 2; *Cyp7a1*, cytochrome P450 family 7 subfamily A polypeptide 1; *Dgat*, diacylglycerol *O*-acyltransferase 1; *Elo1*, fatty acid elongase 1; *Fads1*, delta 5 desaturase/fatty acid desaturase 1; *Fads2*, delta 6 desaturase/fatty acid desaturase 2; *Fasn*, fatty acid synthase; *Gpam*, glycerol-3-phosphate acyltransferase mitochondrial; *Hmgcr*, 3-hydroxy-3-methylglutaryl-CoA reductase; *Hmgcs2*, 3-hydroxy-3-methylglutaryl-CoA synthase 2; *Ldlr*, low density lipoprotein receptor; *Lipc*, lipase hepatic; *Mttp*, microsomal triglyceride transfer protein; *Ppara*, peroxisome proliferator activated receptor alpha; *Scd1*, stearoyl-coenzyme A desaturase 1; *Soat*/*Acat*, sterol-*O*-acyltransferase 1/acyl-CoA:cholesterol acyltransferase; *Sod2*, superoxide dismutase 2, mitochondrial; *Srebf1*, sterol regulatory element binding transcription factor 1.

### 2.4. Effect on Systemic Inflammation

**Figure 4:**
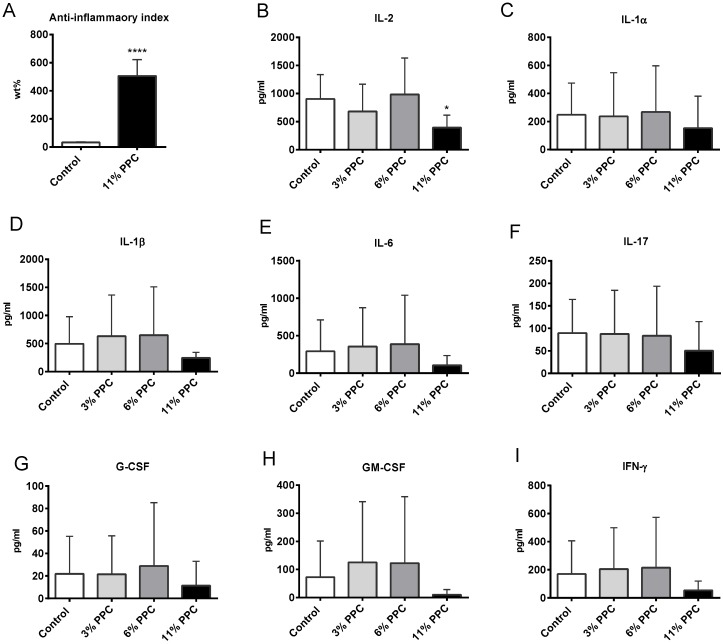
Plasma inflammation parameters in male Wistar rats fed a protein-phospholipid complex (PPC) from krill. (**A**) Fatty acid anti-inflammatory index (defined as ((C20:5*n*-3 + C20:3*n*-6 + C22:6*n*-3 + C22:5*n*-3)/(C20:4*n*-6) × 100)); (**B**) interleukin 2 (IL-2); (**C**) IL-1α; (**D**) IL-1β; (**E**) Il-6; (**F**) IL-17; (**G**) granulocyte colony-stimulating factor (G-CSF); (**H**) granulocyte macrophage colony-stimulating factor (GM-CSF); and (**I**) interferon gamma (IFN-γ). Values are means with standard deviations (*n* = 6). Significant difference from control was determined using unpaired *t*-test (* *p* ≤ 0.05).

PPC also seemed to have an anti-inflammatory potential in plasma, in line with the increased fatty acid anti-inflammatory index (Figure 4A). Cytokine interleukin-2 (IL-2) (Figure 4B) was significantly decreased by the 11% PPC supplemented diet, while PPC tended to reduce plasma levels of IL-1α, IL-1β, IL-6, IL-17, granulocyte colony-stimulating factor (G-CSF), granulocyte macrophage colony-stimulating factor (GM-CSF), and interferon gamma (IFN-γ) (Figure 4C–I). Interestingly, this was only seen at the highest dose of PPC.

### 2.5. Hepatic Fatty Acid Metabolism

**Figure 5:**
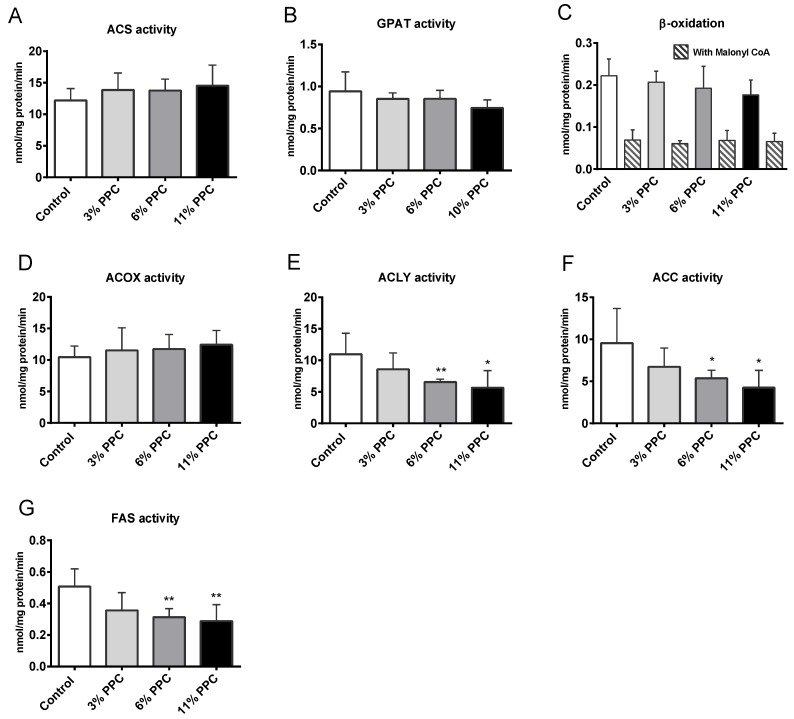
Liver enzyme activity in male Wistar rats fed a protein-phospholipid complex (PPC) from krill. (**A**) Acyl-CoA synthetase (ACS) activity; (**B**) glycerol-3-phosphate acyltransferase (GPAT) activity; (**C**) β-oxidation of palmitoyl-Coenzyme A (CoA) in the absence and presence of malonoyl-CoA inhibitor; (**D**) Acyl-CoA oxidase (ACOX) activity; (**E**) ATP citrate lyase (ACLY) activity; (**F**) Acetyl-CoA carboxylase (ACC) activity; and (**G**) Fatty acid synthase (FAS) activity. Values are means with standard deviations (*n* = 5–6). Significant difference from control was determined using unpaired *t*-test (* *p* ≤ 0.05, ** *p* ≤ 0.01).

The hepatic gene expressions of CD36 (*Cd36*/*Fat*), apolipoprotein B (*ApoB*), arylacetamide deacetylase (Aadac), hepatic lipase (*Lipc*), microsomal TAG transfer protein (*Mttp*), glycero-3-phosphate acyltransferase (*Gpam*) and diacylglycerol *O*-acyltransferase 1 (*Dgat*) were not affected by the 11% PPC feeding compared to controls (Table 2). The acyl-CoA synthetase activity (ACS) was unchanged (Figure 5A), while the glycero-3-phosphate acyltransferase (GPAT) activity tended to decrease by PPC feeding (*p* = 0.08 for 11% PPC *vs*. control, Figure 5B). Hepatic mitochondrial β-oxidation of long-chain fatty acids in the absence and presence of malonyl-CoA was not significantly changed in the PPC-fed groups compared to controls (Figure 5C). The expression of peroxisome proliferator activated receptor, alpha (PPARα) response genes involved in β-oxidation (*Cpt1a* and *Cpt2*), ketone body production (*Hmgcs2*), and acylcarnitine transport (*Cact*/*Slc25a20*), was insignificantly increased, as was *Ppara* mRNA itself (Table 2). Although no increased activity of ACOX was observed at the enzyme level (Figure 5D), *Acox1* mRNA expression was significantly increased in the 11% PPC-fed group compared to controls (Table 2). The activity of ATP-citrate lyase (ACLY) was significantly lower in the 6%- and the 11% PPC-fed group compared to controls (Figure 5E). The same pattern was detected for the activity of acetyl-CoA-carboxylase (ACC) and fatty acid synthase (FAS), with significantly lower activities in the 6%- and in the 11% PPC-fed groups compared to controls (Figure 5F,G). Furthermore, the gene expression of enzymes involved in lipogenesis (*Acaca*, *Fasn* and *Srebf1*) was reduced by the 11% PPC feeding compared to control, however, not significantly (Table 2).

**Figure 6:**
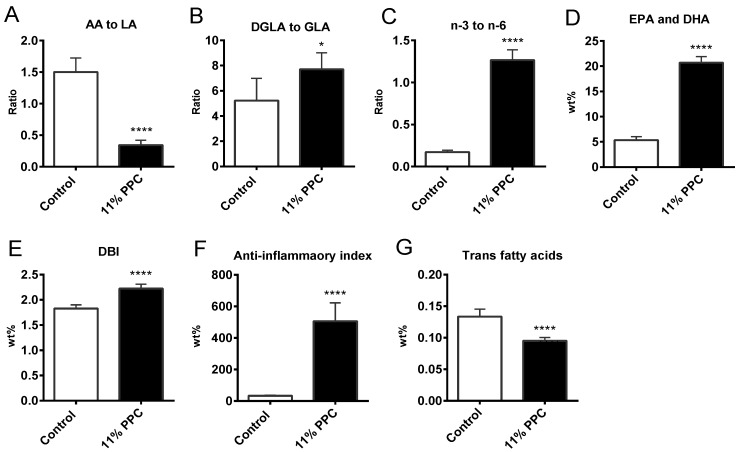
Hepatic fatty acid concentration, ratios and indexes in male Wistar rats fed a protein-phospholipid complex (PPC) from krill for 4 weeks. (**A**) Ratio between AA to LA (C20:4*n*-6 to C18:2*n*-6); (**B**) ratio between dihomo-gamma-linolenic acid (DGLA, C20:3*n*-6) to gamma-linolenic acid (GLA, C18:3*n*-6); (**C**) ratio between *n*-3 to *n*-6 PUFAs; (**D**) wt % of EPA + DHA; (**E**) Double bond index (DBI); (**F**) fatty acid anti-inflammatory index; and (**G**) wt % of *trans* fatty acids. Values are means with standard deviations (*n* = 6). Significant difference from control was determined using unpaired *t*-test (* *p* ≤ 0.05, *** *p* ≤ 0.001).

Hepatic fatty acid composition was determined in controls and in the 11% PPC-fed group (Table 3). The wt % of total SFAs was significantly decreased by dietary PPC compared to controls, mainly due to lower levels of the long chain SFA stearic acid (C18:0) in the PPC-fed group (Table 3). Similarly to the observations in plasma, the wt % of total hepatic MUFAs were not significantly different between controls and the PPC-fed group, nor were the levels of OA (C18:1*n*-9) (Table 3), or the gene expression of Δ9 desaturase (*Scd1*) (Table 2). The level of *n*-9 PUFA was significantly lower in the PPC-fed group, mediated by the level of MA (C20:3*n*-9). As observed in plasma, most *n*-6 PUFAs were reduced by PPC feeding, except for LA and dihomo-gamma-linolenic acid (DGLA, C20:3*n*-6), which led to a 4.4-fold lower ratio of AA to LA (Figure 6A) suggesting decreased activities of the Δ5 and Δ6 desaturases. The reduction in these desaturases was confirmed at the mRNA level (Table 2). Moreover, the ratio of DGLA (C20:3*n*-6) to gamma-linolenic acid (GLA, C18:3*n*-6) increased significantly (Figure 6B), suggesting a diet-induced increased activity of the elongase system. The gene expression of fatty acid elongase 1 (*Elo1*), however, was unchanged by dietary PPC (Table 2). Similar to observations in plasma, PPC feeding increased all hepatic *n*-3 PUFAs (Table 3), the ratio of *n*-3 to *n*-6, the wt % of EPA and DHA, DBI, fatty acid anti-inflammatory index and reduced *trans* fatty acids (Figure 6C–G).

**Table 3:** Fatty acid compositions (wt %) in liver of male Wistar rats ^1^.

	Diets		
	Control	11% PPC
**SFAs**	33.89 ± 0.86	29.31 ± 1.77 ***
C14:0	0.47 ± 0.11	0.62 ± 0.10 *
C16:0	18.42 ± 1.26	18.82 ± 0.91
C18:0	13.62 ± 1.91	8.47 ± 1.23 ***
C20:0	0.05 ± 0.00	0.04 ± 0.00 ***
C22:0	0.12 ± 0.02	0.07 ± 0.01 ***
**MUFAs**	20.06 ± 3.19	20.40 ± 1.71
C16:1*n*-7	2.39 ± 1.02	3.13 ± 0.85
C16:1*n*-9	0.25 ± 0.06	0.27 ± 0.04
C18:1*n*-7	4.08 ± 1.12	3.41 ± 0.69
C18:1*n*-9 (OA)	12.52 ± 1.64	12.81 ± 0.87
C20:1*n*-7	0.11 ± 0.02	0.14 ± 0.01 **
C20:1*n*-9	0.19 ± 0.03	0.12 ± 0.01 ***
C22:1*n*-7	0.01 ± 0.00	0.01 ± 0.00
C22:1*n*-9	0.05 ± 0.03	0.02 ± 0.00
C24:1*n*-9	0.17 ± 0.03	0.13 ± 0.02 *
**PUFAs**	45.91 ± 2.74	50.20 ± 1.30 *
*n*-9 PUFAs	0.17 ± 0.02	0.08 ± 0.00 ***
C20:3*n*-9 (MA)	0.17 ± 0.02	0.08 ± 0.00 ***
*n*-6 PUFAs	39.13 ± 2.74	22.13 ± 1.36 ***
C18:2*n*-6 (LA)	14.80 ± 1.87	15.72 ± 1.40
C18:3*n*-6 (GLA)	0.20 ± 0.03	0.09 ± 0.01 ***
C20:3*n*-6 (DGLA)	1.04 ± 0.38	0.71 ± 0.09
C20:4*n*-6 (AA)	21.96 ± 2.13	5.30 ± 0.91 ***
C22:4*n*-6	0.44 ± 0.06	0.07 ± 0.01 ***
C22:5*n*-6 (DPA)	0.29 ± 0.09	0.05 ± 0.01 ***
*n*-3 PUFAs	6.61 ± 0.61	27.98 ± 2.16 ***
C18:3*n*-3 (ALA)	0.42 ± 0.10	1.47 ± 0.29 ***
C18:4*n*-3	0.02 ± 0.01	0.26 ± 0.08 ***
C20:4*n*-3	0.09 ± 0.01	0.68 ± 0.18 ***
C20:5*n*-3 (EPA)	0.37 ± 0.05	10.82 ± 0.94 ***
C21:5*n*-3 (HPA)	0.00 ± 0.00	0.36 ± 0.07 ***
C22:5*n*-3 (DPA)	0.73 ± 0.09	4.54 ± 0.68 ***
C22:6*n*-3 (DHA)	4.98 ± 0.67	9.85 ± 0.97 ***

^1^ Data were analyzed with *t*-test (*n* = 6), and are presented as means ± SD of wt % (g fatty acids/100 g of total hepatic fatty acids). Values significantly different from control are indicated (* *p* ≤ 0.05, ** *p* ≤ 0.01, *** *p* ≤ 0.001). Abbreviations: See Table 1.

### 2.6. Cholesterol Synthesis, Degradation, and Esterification

In view of the cholesterol-lowering effect observed in the 11% PPC-fed animals, the potential of PPC supplementation to influence the rate-limiting enzymes in cholesterol synthesis, degradation, and esterification was investigated with gene expression analysis. The gene expression of LDL cholesterol receptor (*Ldlr*), 3-hydroxy-3-methyl-glutaryl (HMG)-CoA reductase (*Hmgcr*), cholesterol 7a hydroxylase (*Cyp7a1*) and acyl-CoA:cholesterol acyltransferase (*Soat*/*Acat*) was unchanged (Table 2). 

## 3. Discussion

In the present study we have demonstrated that a phospholipid-protein complex from krill (PPC) has a marked lipid-lowering effect, which may be related to effects on lipid and amino acid metabolic pathways. Only few studies have investigated the mechanism of action of TAG and cholesterol lowering by dietary krill phospholipids and proteins. Moreover, the present data show that PPC has an antioxidative and anti-inflammatory potential.

The present data suggest that TAG lowering in the PPC supplemented diets is mediated through decreased lipogenesis, in line with previous findings of lowered SREBP1c activity after PUFA supplementation [39]. Indeed, PPC feeding resulted in reduced enzyme activities of ACLY, ACC and FAS (Figure 5E–G). Increased fatty acid oxidation, especially the mitochondrial β-oxidation system of long-chain fatty acids, is also related to lowering of plasma levels of TAG by krill oil [40]. However, PPC treatment did not affect the mitochondrial fatty acid oxidation or the production of ketone bodies, as the mitochondrial palmitoyl-CoA oxidation in the presence and absence of malonyl-CoA was unchanged, with insignificantly increased PPARα response on the genes *Cpt1a*, *Cpt2*, *Cact*/*Slc25a20* and *Hmgcs2* (Table 2). The peroxisomal fatty acid oxidation system was probably slightly affected by dietary PPC as the ACOX activity was unaltered (Figure 5D) despite a significantly increased mRNA level (Table 2), and some long-chain SFA shortening observed in plasma and liver. A significantly increased level of plasma C22:1*n*-9 could be a consequence of an increased elongase system. The hepatic gene expression of *Elo1* was, however, unchanged in the PPC treated animals compared to controls (Table 2). The GPAT activity tended to be lowered by the 11% PPC feeding (Figure 5B), although the mRNA levels of both *Gpat* and *Dgat* were unaffected (Table 2). Altogether, the parallel decrease in hepatic lipogenesis and plasma TAG concentration, without any effect on hepatic gene expression of *CD36*/*Fat*, acyl-CoA synthetase (ACS) activity, plasma NEFAs and hepatic mRNA levels of *ApoB* and *Aadac*, suggest that TAG lowering of PPC was linked to hepatic lipogenesis while fatty acid catabolism, transportation, and secretion was unaffected. The activity of lipoprotein lipase in adipose tissue was not measured, but the mRNA level of hepatic lipase (*Lipc*) was unchanged by PPC feeding (Table 2), indicating no increase in the clearance of potential TAG-rich lipoproteins.

The effect on plasma cholesterol levels could be due to a number of factors. Of prime significance is the possibility of reduced cholesterol synthesis and/or degradation. It is well documented that HMG-CoA reductase is rate-limiting in the synthesis of cholesterol under almost all experimental conditions, and inhibition of this enzyme has been shown to reduce plasma cholesterol levels [41]. The 11% PPC diet did, however, not lead to lower gene expression of *Hmgcr* (Table 2). Moreover, the mRNA level of the rate-limiting enzyme in degradation of cholesterol into bile acids, *Cyp7a1*, was unaffected (Table 2), and a decrease in plasma bile acids was seen only in the 3% PPC group (Figure 2F). Furthermore, the gene expression of *Ldlr* and *Soat/Acat* was not affected by dietary PPC (Table 2). The importance of concerted regulation of cholesterol and TAG biosynthesis suggest that further studies are needed to establish whether treatment with PPC affects the metabolic properties of LDL cholesterol particles. 

Interestingly, while krill oil was previously found to reduce the body weight of rats fed high-fat diets for four weeks [42], the PPC diets increased feed efficiency by 17% already at 3% wt % (Figure 1C). This indicates a good digestibility and bioavailability of the protein component of PPC. Furthermore, the higher feed efficiency seen in the PPC-fed animals could be linked to the reduced lipogenesis. The lower feed efficiency in controls indicates higher energy expenditure due to TAG synthesis from glucose (which incur a considerable ATP cost), as hepatic lipogenesis was reduced by 30%–43% in the PPC-fed animals (3% and 11% PPC, respectively). With a relatively low-fat diet, the latter would result in a more energy-efficient use of the PPC feed. Further analysis is necessary to confirm this interpretation. 

Atherosclerosis is a complex vascular disease with a bidirectional interaction between lipids and inflammation as a major feature. Thus, the liver, as a central regulator of fatty acid metabolism and systemic and local inflammatory processes, are involved in the atherosclerotic development. Moreover, research into atherosclerosis has led to many compelling discoveries about mechanisms of the disease, where also the involvement of oxidative stress is considered important in the initiation and progression. Fish consumption is considered health-beneficial as it among others decreases risk of cardiovascular disease by altering the plasma lipid profile, and decreasing inflammation and oxidative stress. In the present study we found liver and plasma levels of *n*-3 PUFAs to increase after PPC supplementation, and in particular the levels of EPA, DHA, DPA and HPA were elevated. The increased wt % of *n*-3 PUFAs was linked to reduced *n*-6 PUFAs, in particular the level of AA, resulting in an increased ratio of *n*-3 to *n*-6 PUFA, as well as the relative level of EPA and DHA. The anti-inflammatory fatty acid index is based on the understanding that EPA and DHA generate anti-inflammatory resolvines, as well as prostaglandins with a lower pro-inflammatory potential than AA, and thus the ratio between these fatty acids will influence inflammatory processes [16]. This index was increased in both plasma and liver (Figure 4 and Figure 6), and linked to a significantly decreased level of plasma IL-2 (Figure 4B). Although the study was performed on young rats on a low-fat diet, PPC tended to reduce a number of cytokines and chemokines, but the data were not statistically significant. Noteworthy, the carotenoid fucoxanthin was shown to suppress the production of inflammatory cytokines including IL1β, IL-6 and tumor necrosis factor-α (TNF-α) in cell studies [43]. Thus, the contribution of astaxanthin towards a lower inflammatory status in the rats fed the PPC supplemented diets should be considered.

Oxidative stress, mainly generated in mitochondria, leads to a decrease in chain length and unsaturation [44]. In the PPC-fed rats, plasma fatty acids had overall longer chain length and increased DBI value (Table 1 and Figure 3F), and the hepatic DBI value was increased (Figure 6E), despite a similar total PUFA-level in the control and PPC diets (Table 3). Moreover, these findings were associated with increased plasma total antioxidant capacity (Figure 3G). The presence of the astaxanthin in PPC could be awarded the oxidative protective status seen in the PPC-fed rats compared to controls. Furthermore, the oxidation of LDL in the vessel wall plays an important role in the development of atherosclerosis, and a high intake of dietary antioxidative carotenoids increases the resistance of LDL oxidation [45]. Thus, the increased plasma and hepatic DBI-value, in addition to the increased plasma antioxidative capacity found in the PPC-fed rats, suggest an additional potential of the PPC as a cardiovascular-protective dietary supplement. 

In conclusion, our observations suggest that supplementation with a phospholipid-protein complex (PPC) from krill can reduce plasma TAG and cholesterol, and results in a more beneficial fatty acid composition in rats, which may suggest an anti-atherogenic potential. Whether antioxidative and anti-inflammatory effects are associated with the content of astaxanthin should be considered. 

## 4. Materials and Methods

### 4.1. Animals and Dietary Interventions

The animal protocol was approved by the Norwegian State Board for Biological Experiments with Living Animals (Approval No. 2013-5324, 23 April 2013), and the experiments were performed in accordance to the Guidelines for the Care and Use of Laboratory Animals and the Guidelines of the Animal Welfare Act. Male Wistar rats, aged five to six weeks (Taconic Tornbjergvej facility, Elby, Denmark), were randomized and housed pair-wise in open cages (*n* = 6 rats per group). They were kept under standard laboratory conditions with temperature 22 ± 1 °C, dark/light cycles of 12/12 h, relative humidity 43% ± 5%, and 20 air changes per hour. The rats were acclimatized under these conditions for one week prior to study start, with free access to standard chow and water. The rats were fed, *ad libitum*, on a 10% fat diet (wt %), either as a control diet (2% soy oil, 8% lard, 20% casein) or an experimental diet, where casein and lard were replaced with PPC at 3%, 6% or 11% (wt %) (Table 4). The diets consisted of bovine casein, lard, soybean oil, cornstarch, dyetrose, sucrose, cellulose fiber, AIN-93-VX vitamin mix, AIN-93GMX mineral mix, l-cystine and choline bitartrate (Dyets Inc., Bethlehem, PA, USA) and *tert*-butyl-hydroquinone (Sigma-Aldrich, Sigma-Aldrich Norway AS, Oslo, Norway). Krill PPC, an Antarctic krill meal from *Euphausia superba* (RIMFROST GENUINE^®^), was delivered by Olympic Seafood AS (Fosnavaag, Norway). The production process of PPC is described in detail in the granted patent [46]. The PPC consisted of 46.4% protein and 45.7% fat (Table 4), and contained 39.0 g phosphatidylcholine, 13 g EPA and 7.9 g DHA per 100 g extracted fat. The fatty acid and amino acid composition of the diets is given in Table 5 and Table 6, respectively. Feed intake and weight gain were determined twice a week.

**Table 4:** Lipid and protein content (wt %) of the experimental diets.

Components ^1^	Diets			
	Control	3% PPC	6% PPC	11% PPC
Lard	8.0	6.5	5.1	3.2
Soy oil	2.0	2.0	2.0	2.0
Casein	23.0	21.5	20.1	18.1
PCC ^2^	-	3.3	6.4	10.9
Lipids from PPC ^3^	-	1.5	2.9	4.8
Protein from PPC	-	1.5	2.9	4.9

^1^ Values shown are wt % (g component/100 g diet) of the pure components in the diets. The diets were isoenergetic and isonitrogenous and contained 20 wt % protein and 10 wt % fat. The energy contribution from fat, carbohydrates, and protein were 22%, 59% and 19%, respectively; ^2^ The phospholipid-protein complex (PPC) consisted of 46.4% crude protein (measured as Nitrogen × 6.25), 45.7% fat, 4.8% ash, 6% moisture and contained 295 mg/kg astaxanthine esters; ^3^ The PPC lipid fraction consisted of 44% triacylglycerols (TAGs), 44.7% phospholipids (PLs), 3.3% non-esterified fatty acids (NEFAs) and 2.3% cholesterol.

### 4.2. Sampling Protocol

After four weeks of diet treatment, fasted rats were anaesthetized by inhalation of 2% isoflurane (Schering-Plough, Kent, UK). The abdomen was opened in the midline and blood was drawn by cardiac puncture in Vacutainer tubes containing 7.5% ethylenediaminetetraacetic acid (EDTA) and immediately chilled on ice for a minimum of 15 min. The samples were centrifuged and plasma was stored at −80 °C prior to analysis. Heart, liver, and adipose tissues (mesenteric, epididymal, perirenal, and subcutaneous white adipose tissue depots) were collected and weighed. A sample from each liver was removed for β-oxidation analysis, while the remaining parts of the liver and the other tissues were immediately snap-frozen in liquid nitrogen and stored at −80 °C until further analysis. 

**Table 5:** Fatty acid composition (wt %) of the experimental diets.

	Diets			
Fatty acids ^1^	Control	3% PPC	6% PPC	11% PPC
**SFAs**	30.9	30.5	27.9	24.7
C14:0	1.0	1.9	2.4	3.4
C16:0	19.5	19.3	18.1	16.4
C18:0	10.2	9.0	7.3	4.8
C20:0	0.1	0.2	0.1	0.1
C22:0	0.1	0.1	<0.1	<0.1
**MUFAs**	35.8	33.4	29.5	23.7
C16:1*n*-7	1.2	1.4	1.5	1.6
C18:1 (*n*-9) + (*n*-7) + (*n*-5)	34.2	31.4	27.5	21.2
C20:1 (*n*-9) + (*n*-7)	0.4	0.5	0.4	0.4
C22:1 (*n*-9) + (*n*-7)	<0.1	0.1	0.1	0.1
C24:1*n*-9	<0.1	<0.1	<0.1	<0.1
**PUFAs**	32.7	32.6	33.4	33.2
***n*-6 PUFAs**	30.3	26.1	23.6	17.7
C18:2*n*-6 (LA)	29.5	25.4	23.2	17.4
C18:3*n*-6	<0.1	<0.1	<0.1	<0.1
C20:2*n*-6	0.5	0.4	0.3	0.2
C20:3*n*-6	0.1	0.1	<0.1	<0.1
C20:4*n*-6 (AA)	0.1	0.1	0.1	0.1
C22:4*n*-6	0.1	0.1	<0.1	<0.1
***n*-3 PUFAs**	2.4	6.5	9.7	15.4
C18:3*n*-3 (ALA)	2.3	2.5	2.8	2.9
C18:4*n*-3	<0.1	0.9	1.7	2.9
C20:3*n*-3	0.1	0.1	<0.1	0.1
C20:4*n*-3	<0.1	<0.1	<0.1	0.1
C20:5*n*-3 (EPA)	<0.1	1.8	3.2	5.7
C21:5*n*-3 (HPA)	<0.1	0.1	0.1	0.2
C22:5*n*-3 (DPA)	<0.1	0.1	<0.1	0.1
C22:6*n*-3 (DHA)	<0.1	1.0	1.9	3.4
*n*-6 PUFAs:*n*-3 PUFAs	12.6:1	4.0:1	2.4:1	1.2:1

^1^ Fat (wt %) Bligh & Dyer. Abbreviations: AA, arachidonic acid; ALA, alpha linolenic acid; DHA, docosahexaenoic acid; DPA, docosapentaenoic acid; EPA, eicosapentaenoic acid; HPA, heneicosapentaenoic acid; LA, linoleic acid; MUFAs, monounsaturated fatty acids; PPC, phospholipid-protein complex; PUFAs, polyunsaturated fatty acids; SFAs, saturated fatty acids.

### 4.3. Quantification of Plasma Parameters

Lipids from plasma were measured enzymatically on a Hitachi 917 system (Roche Diagnostics GmbH, Mannheim, Germany) using the cholesterol (Cholesterol CHOD-PAP, 11491458-216), and triacylglycerol (Triglycerides GPO-PAP, 11730711) kit from Roche Diagnostics, and the free cholesterol (Free Cholesterol FS, Ref 113609910930), non-esterified fatty acid (NEFA FS, Ref 157819910935) and phospholipid kit (Phospholipids FS, Ref 157419910930) from DiaSys (Diagnostic Systems GmbH, Holzheim, Germany). Plasma bile acid was measured enzymatically on a Roche Modular P chemistry analyzer (Roche Diagnostica), using the BA kit (Total Bile Acid Assy Kit, 05471605001) from Diazyme (Diazyme Laboratories, Gregg, CA, USA). The fatty acid composition was determined by GC/MS as previously described [47]. Glucose was measured on Hithachi 917 using the Glucose/HK kit (Roche Diagnostics, Ref 11876899-216). Fasting insulin was measured in two parallels of 10 μL plasma from each rat using a rat/mouse insulin 96 well plate assay ELISA kit (EZRMI-13K) from EMD Millipore (Billerica, MA, USA), according to the manufacturer’s instructions.

**Table 6:** Amino acid composition (wt %) of the experimental diets.

Amino acid ^1^	Diets			
	Control	3% PPC	6% PPC	11% PPC
Aspartic acid	1.32	1.58	1.70	1.74
Glutaminic acid	4.18	4.68	4.65	4.40
Hydroksyproline	<0.10	<0.01	<0.01	<0.01
Serine	1.17	1.25	1.27	1.24
Glycine	0.41	0.44	0.49	0.53
Histidine	0.59	0.61	0.60	0.58
Arginine	0.69	0.78	0.82	0.83
Threonine	0.87	0.91	0.96	0.96
Alanine	0.62	0.70	0.77	0.80
Proline	2.20	2.28	2.23	2.13
Tyrosine	0.83	0.96	0.98	0.98
Valine	1.33	1.43	1.44	1.40
Methionine	0.52	0.58	0.60	0.61
Isoleucine	1.09	1.17	1.21	1.21
Leucine	1.93	2.07	2.14	2.11
Phenylalanine	1.04	1.13	1.17	1.16
Lysine	1.62	1.86	1.80	1.77
Total amino acids detected	19.6	21.5	21.9	21.5

^1^ g amino acids/100 g diet; PPC, phospholipid-protein complex.

### 4.4. Hepatic Enzyme Activities and Fatty Acid Composition

Liver tissue samples were homogenized and a post-nuclear fraction was prepared as previously described [48]. The activities of acyl-CoA synthetase (ACS, EC number 6.2.1.3), ATP-citrate lyase (ACLY, EC number 4.1.3.8), Acetyl-CoA carboxylase (ACC, EC number 6.4.1.2), acyl-CoA oxidase 1, palmitoyl (ACOX1, EC number 1.3.3.6), glycerol-3 phosphate acyltransferase (GPAT, EC number 2.3.1.15) and fatty acid synthase (FAS, EC number 2.3.1.85) were measured in the post-nuclear fraction as described by Skorve *et al.* [49], with some modifications [50]. Palmitoyl-CoA oxidation in the absence and presence of malonyl-CoA was measured in the post-nuclear fraction from liver as acid-soluble products [51]. Total liver fatty acid composition was analyzed in controls and 11% PPC-fed rats as described previously [37].

### 4.5. Gene Expression Analysis

Total cellular RNA was purified from frozen liver samples, and cDNA was produced as described by Vigerust *et al*. [40]. Real-time PCR was performed with Sarstedt 384 well multiply-PCR Plates (Sarstedt Inc., Newton, NC, USA) on the following genes, using probes and primers from Applied Biosystems (Life Technologies Ltd, Paisley, UK): arylacetamide deacylase (*Aadac,* Rn 00571934_m1), acetyl-coenzyme A carboxylase α (*Acaca* Rn00573474), acyl-coenzyme A oxidase 1, palmitoyl (*Acox1* Rn00569216), apoprotein B (*ApoB* Rn01499049_g1), carnitine-acylcarnitine translocase (*Cact/Slc25a20* Rn00588652), carnitine palmitoyltransferase 1A and 2 (*Cpt1a* Rn00580702 and *Cpt2* Rn00563995, respectively), CD36 antigen/fatty acid translocase (*Cd36*/*Fat* Rn00580728), cytochrome P450, family 7, sub-family A, polypeptide 1 (*Cyp7a1* Rn00564065), diacylglycerol *O*-acyltransferase 1 (*Dgat*, Rn00584870_m1), fatty acid elongase 1 (*Elo1*, Rn00592812_m1), delta 5 desaturase/fatty acid desaturase 1 (*Fads1*, Rn00584915_m1), delta 6 desaturase/fatty acid desaturase 2 (*Fads2*, Rn00580220_m1), fatty acid synthase (*Fasn*, Rn00569117_m1), glycerol-3-phosphate acyltransferase mitochondrial (*Gpam*, Rn00568620_m1), HMG-coenzyme A reductase (*Hmgcr*, Rn00585598), HMG-coenzyme A synthase 2 (*Hmgcs2*, Rn00597339), low density lipoprotein receptor (*Ldlr*, Rn00598438), hepatic lipase (*Lipc*, Rn01530834_m1), microsomal triacylglycerol transfer protein (*Mttp*, Rn01522963_m1), peroxisome proliferator activated receptor, alpha (*Pparα*, Rn00566193), stearoyl-coenzyme A desaturase 1 (*Scd1*, Rn00594894_g1), sterol-*O*-acyltransferase 1/acyl-CoA:cholesterol acyltransferase (*Soat*/*Acat*, Rn00579605), superoxide dismutase 2, mitochondrial (*Sod2*, Rn00690588_g1), sterol regulatory element binding factor 1 (*Srebf1*, Rn01495769_m1).

Three different reference genes were included: Eukaryotic 18S ribosomal RNA (*18S*, Kit-FAM-TAMRA (Reference RT-CKFT-18s) from Eurogentec, Seraing, Belgium), glyceraldehyde-3-phosphate dehydrogenase (*Gapdh*, Mm99999915_g1, from Applied Biosystems), Ribosomal protein, large, P0 (*Rplp0*, catalog no. 4333761T, from Applied Biosystems). Data normalized to *Rplp0* are presented.

### 4.6. Total Antioxidant Capacity and Inflammatory Markers

Total antioxidant capacity of plasma was measured using the total antioxidant capacity kit (Abcam, Cambridge, UK) according to the manufacturer’s instructions. The protein mask was not used, enabling the analysis of both small molecule antioxidants and proteins capacity to reduce Cu^2+^ to Cu^+^. In brief, EDTA-plasma was allowed to reduce Cu^2+^ for 1.5 h, at room temperature, on an orbital shaker. The absorbance was measured at 570 nm using a plate reader. Results were expressed as trolox equivalent according to a trolox standard curve.

The levels of IL-1α, IL-1β, IL-2, IL-6, IL-17, G-CSF, GM-CSF and INF-γ were measured in plasma samples using a custom-made multiplex MILLIPLEX MAP kit (Millipore Corp., St. Charles, IL, USA), and the assay solution was read by the Bio-Plex array reader (Bio-Rad, Hercules, CA, USA) and determined with the Bio-Plex Manager Software 4.1.

### 4.7. Statistical Analysis

Data sets were analyzed using Prism Software (Graph-Pad Software, version 6, San Diego, CA, USA) to determine statistical significance. The results are reported as means of 5–6 animals per group with their standard deviations (SD). Normal distribution of samples was analyzed using Kolmogorov-Smirnov test with Dallal-Wilkinson-Lillie for *p* value. An unpaired *t*-test was performed to evaluate statistical differences between groups. *p*-Values ≤0.05 were considered significant.

## Supplementary Files

Supplementary File 1
