# Supplementary material for: A Phospholipid-Protein Complex from Krill with Antioxidative and Immunomodulating Properties Reduced Plasma Triacylglycerol and Hepatic Lipogenesis in Rats
Source: Mar Drugs. 2015 Jul 16;13(7):4375–97. doi: 10.3390/md13074375 (PMC4515623; doi:10.3390/md13074375)
Supplement: Supplementary File 1 [file marinedrugs-13-04375-s001.docx]

Supplementary Information

**Figure S1.** Weight of liver, heart and different adipose tissue depots. PPC,
phospholipid-protein complex.

**Figure S2.** Plasma levels of high-density lipoprotein (HDL)-cholesterol, low-density lipoprotein (LDL)-cholesterol, cholesterol, non-esterified fatty acid (NEFA), insulin and glucose. PPC, phospholipid-protein complex.

© 2015 by the authors; licensee MDPI, Basel, Switzerland. This article is an open access article distributed under the terms and conditions of the Creative Commons Attribution license (http://creativecommons.org/licenses/by/4.0/).
